# The Story of the Insane from Year to Year

**Published:** 1900-01-13

**Authors:** 


### THE STORY OF THE INSANE FROM YEAR TO YEAR.

Twelfth Series.

### NEWCASTLE-UPON-TYNE ASYLUM.

At the beginning of the year 1898 this asylum contained 503 patients, and by the end of December the numbers had risen to 516. There were 120 first admissions, 18 readmissions, 43 recoveries, 15 discharged relieved, and 67 deaths. Calculated on the admissions the recoveries stand at 31·16 per cent., and the deaths were 13 per cent. on the average number resident. Of these deaths 37 were caused by brain diseases, 6 by consumption, and 3 by pneumonia. Twelve of the year's admissions owed their insanity to such moral causes as adverse circumstances, 20 to intemperance in drink, and 15 to hereditary influence. We have more than once warned our readers not to accept the percentages of recoveries in any one year as a criterion of the management of an asylum, and the statistics of this asylum shows how worthless such a course would be unless all the attending circumstances were duly considered. Here the recoveries on the male side were only 19·48 per cent. of the admissions, while those on the female side were 45·9 per cent., although climate, diet, treatment, &c., are as nearly as possible identical. The committee say they have "much pleasure in again expressing their entire satisfaction with Dr. Callcott and his staff in the general discharge of their duties." The weekly cost of maintenance is 9s. per head.

